# Immune Tolerance Induction (ITI) with a pdFVIII/VWF Concentrate (octanate) in 100 Patients in the Observational ITI (ObsITI) Study

**DOI:** 10.1055/s-0042-1748756

**Published:** 2022-05-26

**Authors:** Carmen Escuriola Ettingshausen, Vladimír Vdovin, Nadezhda Zozulya, Pavel Svirin, Tatiana Andreeva, Majda Benedik-Dolničar, Victor Jiménez-Yuste, Lidija Kitanovski, Silva Zupancic-Šalek, Anna Pavlova, Angelika Bátorová, Cesar Montaño Mejía, Gulnara Abdilova, Sigurd Knaub, Martina Jansen, Shannely Lowndes, Larisa Belyanskaya, Olaf Walter, Johannes Oldenburg, on behalf of the ObsITI Study Group

**Affiliations:** 1 HZRM, Hämophilie-Zentrum Rhein Main, Mörfelden-Walldorf, Germany; 2 Morozovskaya Children's Hospital, Moscow, Russian Federation; 3 National Research Center for Hematology, Moscow, Russian Federation; 4 City Center for the Treatment of Hemophilia Patients, City Polyclinic N° 37, St. Petersburg, Russian Federation; 5 Children's Hospital Oncology-Hematology Unit, University Medical Center Ljubljana, Ljubljana, Slovenia; 6 Servicio de Hematología, Hospital Univeristario La Paz, Autónoma University, Madrid, Spain; 7 Division of Haematology, Haemophilia Centre, University Hospital REBRO, Zagreb, Croatia; 8 Institute of Experimental Haematology and Transfusion Medicine, University Clinic Bonn, Bonn, Germany; 9 Department of Hematology and Transfusion Medicine, National Hemophilia Center, Faculty of Medicine of the Comenius University and University Hospital, Bratislava, Slovak Republic; 10 Hemolife and Universidad Tecnológica de Pereira, Pereira, Colombia; 11 Scientific Center of Pediatrics and Pediatric Surgery, Almaty, Kazakhstan; 12 Octapharma AG, Lachen, Switzerland; 13 Octapharma Pharmazeutika Produktionsges.mbH, Vienna, Austria

**Keywords:** factor VIII, von Willebrand factor, FVIII inhibitors, hemophilia A, immune tolerance

## Abstract

**Background** Immune tolerance induction (ITI) with repeated factor VIII (FVIII) administration is the only strategy proven to eradicate inhibitors. The observational ITI study is evaluating ITI with a range of FVIII products.

**Methods** This subgroup analysis reports prospective interim data for patients treated with a plasma-derived, von Willebrand factor-stabilized FVIII concentrate (pdFVIII/VWF, octanate). Complete success (CS) of ITI required achievement of three criteria: inhibitor titer < 0.6 BU/mL; FVIII recovery ≥ 66%; FVIII half-life ≥6 hours. Partial success (PS) required achievement of two criteria and partial response (PR) one. ITI success was defined as CS or PS. Data were analyzed for patients who achieved CS, had 36 months' observation, or failed ITI.

**Results** One-hundred prospectively enrolled patients were included in the analysis; 91 had poor prognosis factors for ITI success. The mean (standard deviation) daily ITI dose was 116.4 (61.1) IU FVIII/kg in 14 low responders (< 5 BU/mL) and 173.7 (112.0) IU FVIII/kg in 86 high responders (≥ 5 BU/mL). Inhibitor titers < 0.6 BU/mL were achieved in 71% of patients in a median of 4.01 months, accompanied by a 93% reduction in bleeding rate. ITI success was achieved by 70% of patients and 56 of 72 (78%) primary (first-line) ITI patients. PR was achieved by 5 patients; ITI failed in 25 patients. PS and CS were achieved in a median of 5.55 and 11.25 months, respectively.

**Conclusions** ITI with pdFVIII/VWF led to rapid eradication of FVIII inhibitors, normalization of FVIII pharmacokinetics in the majority of patients, and a significant reduction in bleeding rates.

## Introduction

Hemophilia A treatment with factor VIII (FVIII) replacement continues to be limited by FVIII inhibitor development in ∼25 to 40% of previously untreated patients. 1 Development of a FVIII inhibitor reduces the effectiveness of FVIII replacement to prevent and manage bleeding and results in increased morbidity, mortality, and socioeconomic burden. 2 3 Inhibitor development is linked to multiple factors, some inherent to the patient (e.g., *F8* gene mutation, immune factors, ethnicity, familial predisposition) and some environmental (e.g., treatment intensity, FVIII product, inflammation/infection). 4 5 6

Inhibitor patients are classified as high responders or low responders. 7 High responders produce a rapid anamnestic response to exogenous FVIII reaching inhibitor titers ≥ 5 Bethesda units (BU)/mL, while low responders display titers that never exceed 5 BU/mL. 8 Immune tolerance induction (ITI), which involves prolonged treatment with plasma-derived (pdFVIII) or recombinant FVIII (rFVIII), is the only clinically proven strategy for inhibitor eradication, 1 9 and is recommended as the primary treatment option in European and US guidelines. 9 10 11 12

Poor prognosis factors for ITI success include age ≥ 7 years at ITI initiation, ITI delay ≥ 2 years since inhibitor diagnosis, historical inhibitor titer ≥ 200 BU/mL, inhibitor titer ≥ 10 BU/mL at ITI initiation, peak inhibitor titer during ITI, and prior ITI failure. 13 14 15 It has been suggested that ITI treatment success rates might be higher in patients treated with pdFVIII containing von Willebrand factor (pdFVIII/VWF) compared with rFVIII, 16 17 18 potentially due to epitope masking and protection of FVIII from endocytosis by antigen-presenting cells. 18 19

The observational ITI (ObsITI) is an ongoing study that is evaluating the efficacy and safety of ITI in hemophilia A patients with inhibitors and potential predictors of ITI outcome and morbidity. The ObsITI study is the largest investigator-initiated, observational study systematically documenting patients with hemophilia A and inhibitors undergoing ITI with a range of FVIII products. Interim data were published previously for a subgroup of 48 patients in the ObsITI study treated exclusively with a single pdFVIII/VWF concentrate (octanate) administered mainly (67%) using the Bonn protocol, using stringent ITI success criteria (inhibitor titer < 0.6 BU/mL, FVIII recovery ≥ 80%, and FVIII half-life ≥ 7 hours). 14 Inhibitors were not detectable in 38 of 48 (79%) patients in a median time of 3.9 months, and 34 (71%) patients achieved all three criteria in a median time of 10.9 months. 14

Here, we report an updated analysis on 100 patients prospectively enrolled in the ObsITI study who were treated with pdFVIII/VWF during ITI.

## Methods

### Study Design and Patients

The overall design of the study (NCT02207894) has been described previously. 14 Briefly, ObsITI is an ongoing noninterventional study to evaluate the impact of treatment and patient variables on ITI outcomes. ObsITI is open to ITI patients treated with any FVIII product. Product choice and treatment regimen for ITI are at the investigator's discretion; however, the Bonn protocol 20 is the recommended ITI regimen. The study is coordinated by HZRM Haemophilia Centre Rhine-Main, Germany, and has been approved by the relevant ethics committees.

Participants are males of any age with hemophilia A and a clinically relevant FVIII inhibitor, that is, titers of ≥ 0.6 BU/mL and/or with reduced FVIII recovery or half-life. Informed consent and an ability to comply with the study protocol are required for inclusion in the study. Patients with historically defined “poor prognosis factors” for ITI success (e.g., age ≥ 7 years at ITI start, ITI delay ≥ 2 years, historical peak titer ≥ 200 BU/mL, inhibitor titer ≥ 10 BU/mL at ITI start, failed prior ITI attempt) are eligible for the study. Patients with congenital or acquired bleeding defects other than hemophilia A, with concomitant immunological diseases, or with a history of hypersensitivity to blood products and/or FVIII concentrates are excluded. Patients receiving immunosuppressive treatment combined with FVIII for ITI can be included.

The present analysis reports interim data for a subgroup of prospectively enrolled patients treated exclusively with a single pdFVIII/VWF (octanate; Octapharma, Lachen, Switzerland) between December 2005 and January 2019. The analysis population included patients who completed either ≥ 36 months of study or who achieved complete success (CS) or terminated the study prematurely. Patients switching from this pdFVIII/VWF to alternative products during ITI were included in the analysis up to the point that the switch to an alternative product occurred.

### FVIII Inhibitor Titers and FVIII Plasma Levels

FVIII inhibitor titers, FVIII recovery, and FVIII half-life were measured at local laboratories during routine FVIII treatment with no washout period using previously described methods. 14 If no local laboratory measurements were available, the results of the central laboratory (HZRM), if available, were used. At ITI start, FVIII inhibitor titers were measured up to twice monthly using the Bethesda assay (Nijmegen modification). 21 FVIII recovery and FVIII half-life were determined by measurement of FVIII plasma levels. Recommended time points for FVIII recovery determinations were pretreatment and at 15 or 30 minutes after administration of FVIII. Recommended time points for FVIII half-life determinations were pretreatment and at 15 or 30 minutes, and 1, 2, 4, 8, and 12 or 24 hours after administration of FVIII. Recovery was determined at the earliest if inhibitor titer decreased to < 5 BU/mL for the first time and repeated during the ITI course. Half-life determination was performed at the earliest if inhibitor titer was < 0.6 BU/mL for the first time, but preferably after normalized recovery was also confirmed. Final half-life determination was recommended when a regular prophylactic treatment schedule was achieved and following a washout period of 48 to 72 hours.

### ITI Treatment Regimen

The Bonn protocol was recommended as the ITI treatment regimen of choice. 20 Patients starting ITI according to the Bonn protocol received 50 to 100 IU FVIII/kg daily or every other day (low responders), or 100 IU FVIII/kg every 12 hours (high responders). The dose was classified as high dose if it was ≥ 150 IU FVIII/kg/day for high responders or ≥ 50 IU FVIII/kg/day or every 2 days for low responders; lower doses were classified as low dose. Upon achievement of CS or partial success (PS), FVIII dose was slowly tapered down every 6 to 8 weeks to ≤ 50 IU FVIII/kg every second day as prophylaxis in all patients.

Bypassing agents (BPAs; recombinant activated factor VII [rFVIIa] or activated prothrombin complex concentrate [aPCC]) were additionally administered in cases of increased bleeding tendency or acute bleeding during ITI. The recommended rFVIIa dose was 90 to 120 μg/kg every 2 hours, or a single dose up to 270 μg/kg, depending on the severity of hemorrhage. An aPCC dose of 75 to 100 U/kg, twice daily, was recommended depending on severity of hemorrhage. Sequential or combined therapy with aPCC and rFVIIa could be considered in cases of an insufficient effect with aPCC and/or rFVIIa. For prophylactic aPCC treatment in patients with a high bleeding tendency, a dose of 50 U/kg one to two times daily was recommended until an inhibitor titer of < 2 BU/mL was achieved or FVIII was measurable again.

### ITI Outcome Definitions

The ITI outcome definitions specified in the protocol of the ObsITI study were inhibitor titer < 0.6 BU/mL, FVIII recovery ≥ 80%, and FVIII half-life ≥ 7 hours. However, since the study started in 2005, this definition has been largely replaced by the definitions used in the international ITI (I-ITI) study (inhibitor titer < 0.6 BU/mL, FVIII recovery ≥ 66% and FVIII half-life ≥ 6 hours), 15 which have been widely used in clinical studies and recommended in treatment guidelines. 10 15 22 23 24 25 ITI outcomes from this interim analysis are reported using the protocol definition as the primary outcome, as well as using the I-ITI definition to allow comparison of the data with other published or ongoing studies of ITI. ITI outcome was defined on a cumulative basis, according to achievement of criterion I (inhibitor titer < 0.6 BU/mL), criterion II (FVIII recovery ≥ 80% or ≥ 66% of the predefined reference value of 1.5% IU/kg ≤ 1 hour post-injection), and criterion III (FVIII half-life ≥ 7 hours or ≥ 6 hours). CS required achievement of all three criteria, PS required achievement of two and partial response (PR) required achievement of one of the three criteria. ITI success in this analysis was defined as CS or PS, that is, at least two of the three criteria were met. Time to tolerization was calculated for patients who achieved ITI success criteria at any time in the study. Relapse monitoring was performed over 12 months using the Bethesda assay and FVIII:C trough levels during regular FVIII prophylaxis reflecting normal FVIII half-life. Relapse was defined as the reappearance of an inhibitor titer ≥ 0.6 BU/mL at two consecutive time points within 12 months of achieving CS or PS and having reached the aimed prophylactic treatment regimen.

Bleeding episodes (BEs), use of BPAs, adverse drug reactions (ADRs), and infections and vaccinations potentially affecting ITI were recorded throughout the study period.

### Statistical Analysis

The ObsITI study plans to enroll 300 patients (at least 200 patients treated prospectively and a maximum of 100 retrospectively). As the choice of therapy is at the investigator's discretion, the number of patients treated with a single pdFVIII/VWF concentrate and assessed in the present analysis was not predetermined.

All statistical analyses were performed using SAS for Windows (Version 9.3 or higher). Descriptive statistics are presented for continuous and categorical data. All statistical tests are two-sided with a type I error ( *p* value) probability of α = 0.05. All confidence intervals are two-sided with confidence probability of 1-α = 0.95. The association of various factors with ITI outcome and time to complete ITI success were assessed using the Cochran-Mantel-Haenszel test and the Log-rank test, respectively.

## Results

### Patient Characteristics

At the time of this analysis (January 2019), a total of 209 patients had been enrolled and treated with various rFVIII and pdFVIII/VWF concentrates. Of these, 100 prospectively enrolled patients at 43 centers in 17 countries had completed ITI with a single pdFVIII/VWF concentrate (octanate) and were included in the subgroup analysis, including 48 patients from a previously published interim analysis. 14 Eighty-six patients were high responders and 14 were low responders. Table 1 shows demographics and baseline clinical characteristics of all patients and according to responder status. At least one risk factor historically associated with a poor ITI prognosis was present in 91 patients.

**Table 1:** Patient demographics and baseline clinical characteristics

Parameter	Low responders ( *n* = 14)	High responders ( *n* = 86)	All patients ( *n* = 100)
Caucasian, *n* (%)	12 (85.7)	61 (70.9)	73 (73.0)
Hemophilia A severity, *n* (%)			
Severe (FVIII:C ≤ 1%)	12 (85.7)	74 (86.0)	86 (86.0)
Moderate (> 1% FVIII:C ≤ 5%)	2 (14.3)	12 (14.0)	14 (14.0)
*F8* mutation a , *n* (%)			
Intron 22 inversion	6 (66.7)	33 (45.8)	39 (48.2)
Intron 1 inversion	0	1 (1.4)	1 (1.2)
Large deletion	1 (11.1)	9 (12.5)	10 (12.4)
Nonsense mutation	0	14 (19.4)	14 (17.3)
Splice site mutation	0	2 (2.8)	2 (2.5)
Small deletion	2 (22.2)	9 (12.5)	11 (13.6)
Small insertion	0	2 (2.8)	2 (2.5)
Missense mutation	0	2 (2.8)	2 (2.5)
Age at inhibitor detection, years, median (IQR)	3.62 (1.82–7.23)	2.27 (1.42–5.19)	2.39 (1.47–5.38)
Age at start of ITI, years, median (IQR)	10.80 (4.25–20.71)	5.38 (2.92–14.11)	5.58 (2.99–15.13)
≤ 12 years, *n* (%)	7 (50.0)	62 (72.1)	69 (69.0)
13 to 17 years, *n* (%)	3 (21.4)	8 (9.3)	11 (11.0)
≥ 18 years, *n* (%)	4 (28.6)	16 (18.6)	20 (20.0)
Inhibitor titer at ITI start, BU/mL, mean (SD)	1.14 (1.15)	339 (1722)	291 (1600)
Patients with ≥ 1 poor prognosis factor, *n* (%)	10 (71.4)	81 (94.2)	91 (91.0)
Previous ITI treatment, *n* (%)	3 (21.4)	25 (29.1)	28 (28.0)
ITI start ≥ 2 years after inhibitor development	7 (50.0)	40 (46.5)	47 (47.0)
Age ≥ 7 years at ITI initiation	8 (57.1)	29 (33.7)	37 (37.0)
Inhibitor titer at ITI start ≥ 10 BU/mL	0	75 (87.2)	75 (75.0)

Abbreviations: FVIII, factor VIII; IQR, interquartile range; ITI, immune tolerance induction; SD, standard deviation.

a *F8* mutation data were available for 81 patients (9 low responders and 72 high responders).

High responders received a mean (standard deviation [SD]) daily dose of 174 (112) IU FVIII/kg and low responders received a mean (SD) dose daily of 116 (61) IU FVIII/kg. Fifty-nine patients started ITI on a high-dose or Bonn protocol (≥ 150 IU FVIII/kg/day at ITI start for high responders, and ≥ 50 IU FVIII/kg either daily or every other day at ITI start for low responders); the remaining 41 started on lower doses and frequencies ( Supplementary Table S1 ). Corresponding data for patients who did or did not achieve ITI success are provided in Supplementary Tables S2 and S3 , respectively. The mean (SD) doses in patients who did or did not achieve success were 155 (109) and 192 (103) IU FVIII/kg/day, respectively. Low responders and high responders were treated for a mean (SD) of 843 (568) and 851 (485) exposure days, respectively.

### ITI Success Rates

**Fig. 1:**
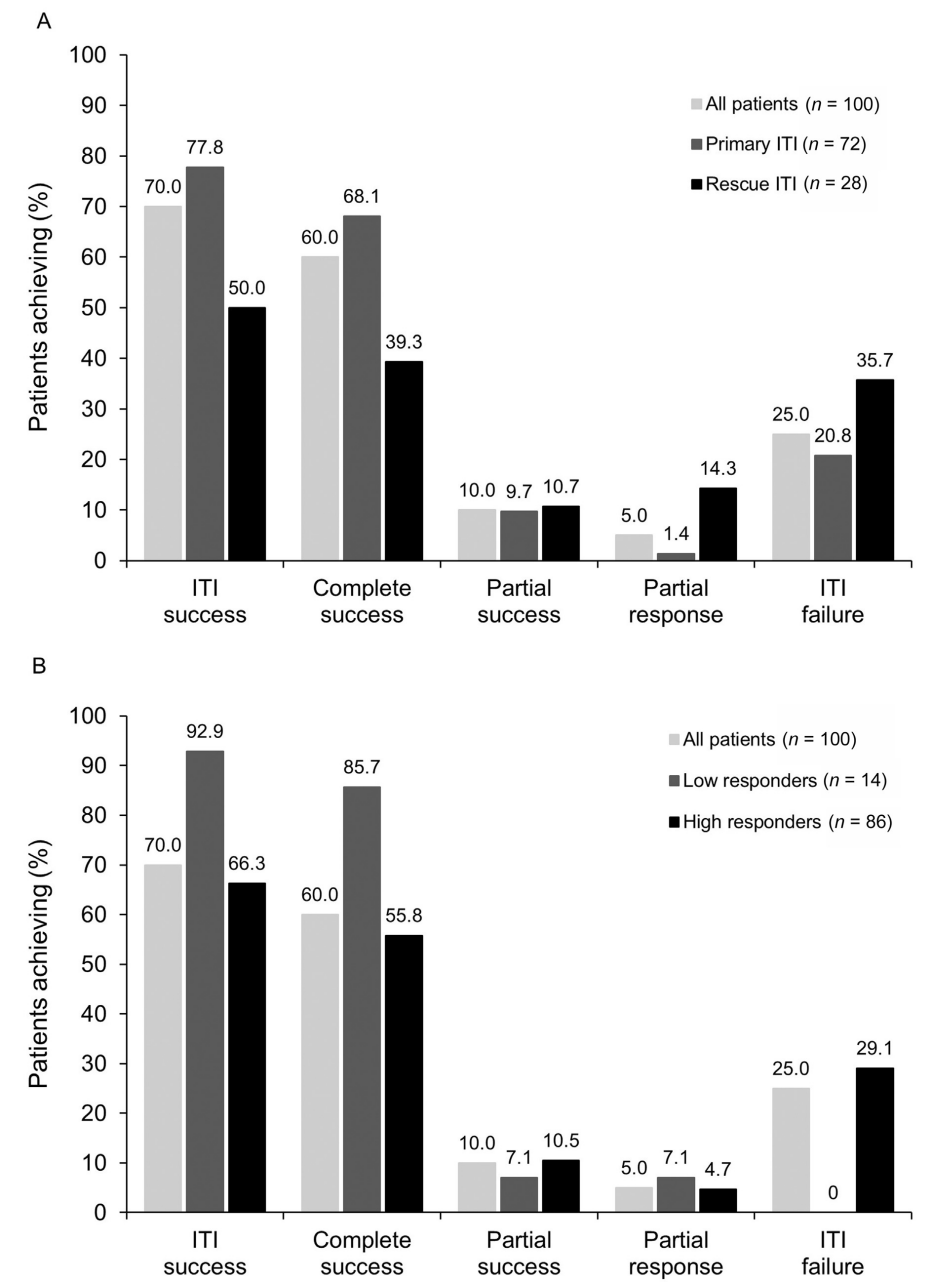
Achievement of immune tolerance induction (ITI) outcomes according to primary or rescue ITI ( **A** ) and low or high responders ( **B** ).

The ITI success rate based on the protocol-defined criteria (inhibitor titer < 0.6 BU/mL, FVIII recovery ≥ 80% and FVIII half-life ≥ 7 hours) in 97 patients was 69.1% (56.7% CS and 12.4% PS). ITI success based on I-ITI criteria (inhibitor titer < 0.6 BU/mL, FVIII recovery ≥ 66% and FVIII half-life ≥ 6 hours) was achieved by 70 of 100 (70.0%) patients (60.0% CS and 10.0% PS), 5.0% of patients achieved PR, and ITI failed in 25.0% of patients ( Fig. 1 ).

ITI success was achieved by 77.8% of primary ITI patients and 50.0% of rescue ITI patients ( Fig. 1A ), by 92.9% of low responders and 66.3% of high responders ( Fig. 1B ). ITI success was achieved by 88.9% of patients without poor prognostic factors and 68.1% of patients with ≥ 1 poor prognostic factor.

**Fig. 2:**
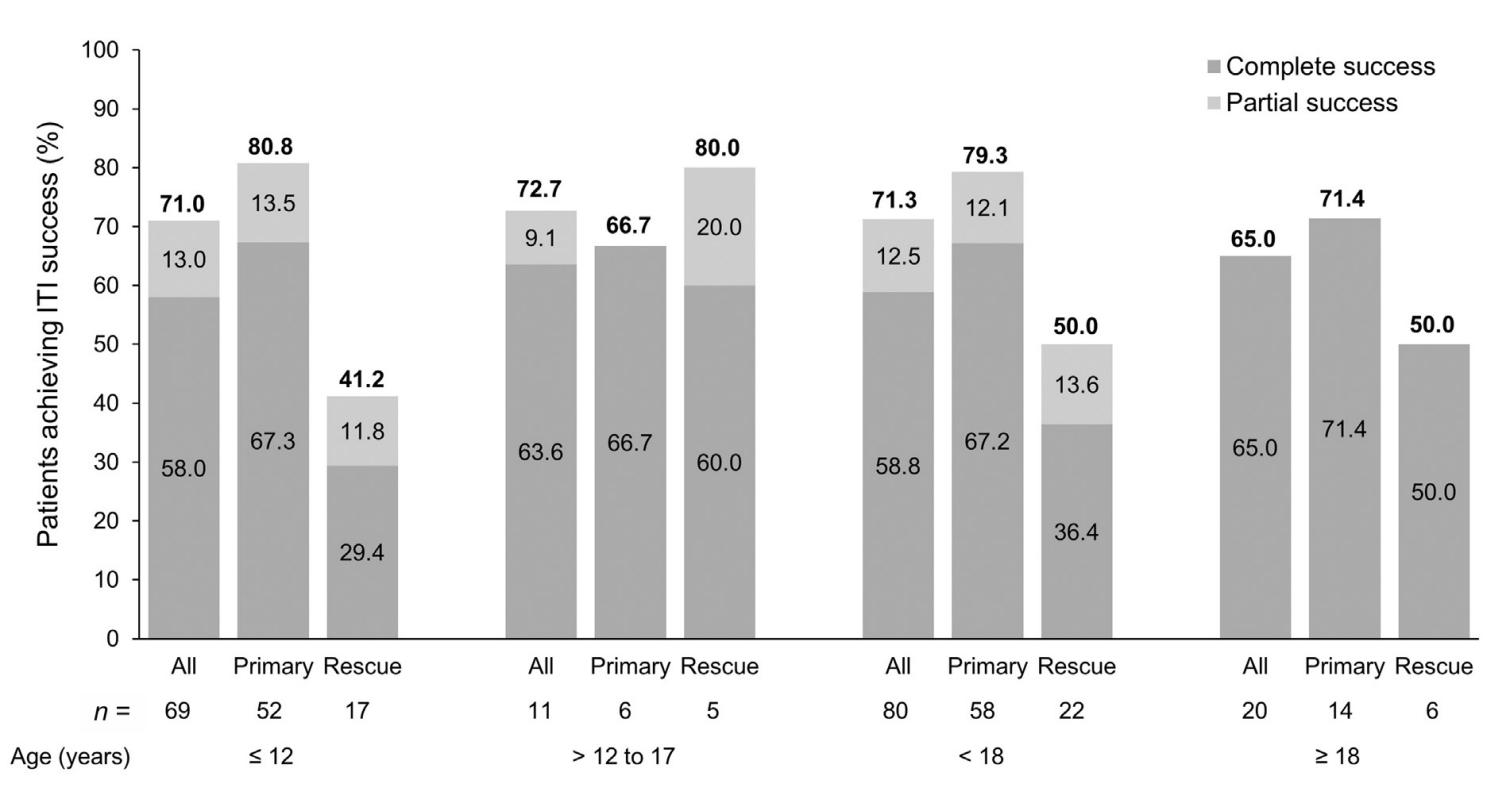
Achievement of immune tolerance induction (ITI) success according to age groups and prior ITI treatment (primary vs. rescue ITI).

ITI success was achieved in 71.3% of patients < 18 years and 65.0% of patients ≥ 18 years ( Fig. 2 ). In primary ITI patients, ITI success was achieved by 79.3% of patients < 18 years and 71.4% of patients ≥ 18 years; the highest ITI success rate was achieved in primary ITI patients ≤ 12 years (80.8%) ( Fig. 2 ).

In a univariate analysis, the following factors were associated with significantly improved ITI outcome: primary ITI (vs. rescue ITI, *p* = 0.012); low responder (vs. high responder, *p* = 0.022); lower number of poor prognostic factors ( *p* = 0.015); and baseline inhibitor titer < 10 BU/mL (vs. ≥ 10 BU/mL, *p* = 0.002). The following factors were not significantly associated with ITI success: time to ITI from inhibitor detection < 2 years (vs. ≥ 2 years, *p* = 0.31); age at start of ITI < 7 years (vs. ≥ 7 years, *p* = 0.67); low ITI dose (vs. high dose, *p* = 0.93); or *F8* mutation type (intron 22/intron 1 inversion vs. large deletions vs. other mutations, *p* = 1.00). In a multiple logistic regression analysis, primary ITI (vs. rescue ITI, *p* = 0.021) and baseline inhibitor titer ≤ 10 BU/mL (vs. > 10 BU/mL, *p* = 0.006) were still associated with a significantly improved ITI outcome.

No patients relapsed within 12 months of achieving CS. One high responder who achieved PS was lost to follow-up. This patient had four poor prognosis factors and had failed four previous ITI attempts with other products (Fanhdi/Grifols, Kogenate/Bayer, Fanhdi + immunoglobulin G [IgG], and Fanhdi + rituximab + IgG). ITI with pdFVIII/VWF (100 IU FVIII/kg/day) and aPCC (FEIBA) prophylaxis were started in April 2010 at 13 years of age. In September 2013, he switched to a prophylactic dose of < 50 IU FVIII/kg administered three times per week. Positive inhibitor tests were recorded in February 2015 (2.7 BU/mL) and March 2015 (0.9 BU/mL) and rituximab was added in March 2015. The final inhibitor test taken in May 2015 was negative and PS was achieved at this point. The patient was lost to follow-up in October 2017. The patient had two treated BEs prior to achieving PS and two treated BEs after achieving PS up to October 2017.

### Time to ITI Success

Times to achievement and interquartile range [IQR] for each of the three ITI success criteria are shown in Table 2 . Negative inhibitor titers were achieved in a median of 4.01 months overall: 4.01 in primary ITI patients, 4.80 in rescue ITI patients, 0.82 in low responders, and 4.17 in high responders.

**Table 2:** Time-to-achievement of ITI success according to responder type and prior ITI

ITI success criteria	Patients	Median (IQR) time to achievement of criteria, months		
		Low responders	High responders	All
FVIII inhibitor titer < 0.6 BU/mL ( *n* = 71)	All patients	0.82 (0.30–7.23)	4.17 (2.14–8.97)	4.01 (1.61–8.97)
	Primary ITI	2.84 (0.26–7.23)	4.01 (2.14–8.97)	4.01 (1.61–8.97)
	Rescue ITI	0.82 (0.72–12.65)	5.72 (2.53–7.16)	4.80 (1.30–9.45)
FVIII recovery ≥ 66% ( *n* = 72)	All patients	2.50 (0.69–3.94)	8.97 (3.42–15.57)	5.82 (2.92–13.75)
	Primary ITI	2.53 (0.69–3.94)	9.03 (3.45–16.59)	6.36 (2.92–15.36)
	Rescue ITI	0.72 (0.13–7.23)	5.32 (3.22–10.84)	5.29 (1.95–10.84)
FVIII half-life ≥ 6 hours ( *n* = 62)	All patients	6.47 (4.93–12.35)	10.87 (5.98–20.53)	10.12 (5.75–16.79)
	Primary ITI	6.24 (2.33–16.79)	11.40 (5.98–21.62)	8.15 (5.49–21.19)
	Rescue ITI	12.29 (9.40–12.35)	10.86 (5.68–13.54)	10.87 (8.18–12.35)
Partial success ( *n* = 70)	All patients	2.33 (0.69–6.11)	7.13 (3.68–15.57)	5.55 (3.19–12.48)
	Primary ITI	2.41 (0.26–6.11)	7.44 (3.68–16.59)	5.75 (3.15–15.36)
	Rescue ITI	0.82 (0.72–9.40)	5.72 (3.22–11.73)	5.49 (3.19–10.84)
Complete success ( *n* = 60)	All patients	11.91 (6.42–16.92)	10.96 (6.67–18.56)	11.25 (6.52–18.56)
	Primary ITI	10.09 (6.37–21.19)	11.25 (6.49–24.87)	11.04 (6.41–21.62)
	Rescue ITI	12.35 (12.29–12.65)	10.86 (7.47–13.54)	12.19 (8.18–12.65)

Abbreviations: FVIII, factor VIII; IQR, interquartile range; ITI, immune tolerance induction.

PS was achieved in a median of 5.55 months and CS in a median of 11.25 months ( Table 2 ). In primary ITI patients, PS was achieved in 5.75 months and CS was achieved in 11.04 months. In rescue ITI patients, PS was achieved in 5.49 months and CS was achieved in 12.19 months.

In children (< 18 years), PS was achieved in a median time of 7.10 months (IQR: 3.42–15.15) in 57 children and CS was achieved in a median time of 10.84 months (IQR: 6.41–15.15) in 47 children. In 13 adults, the median times to PS and CS were 3.91 months (IQR: 0.46–6.24) and 12.65 months (IQR: 9.69–21.19), respectively.

**Fig. 3:**
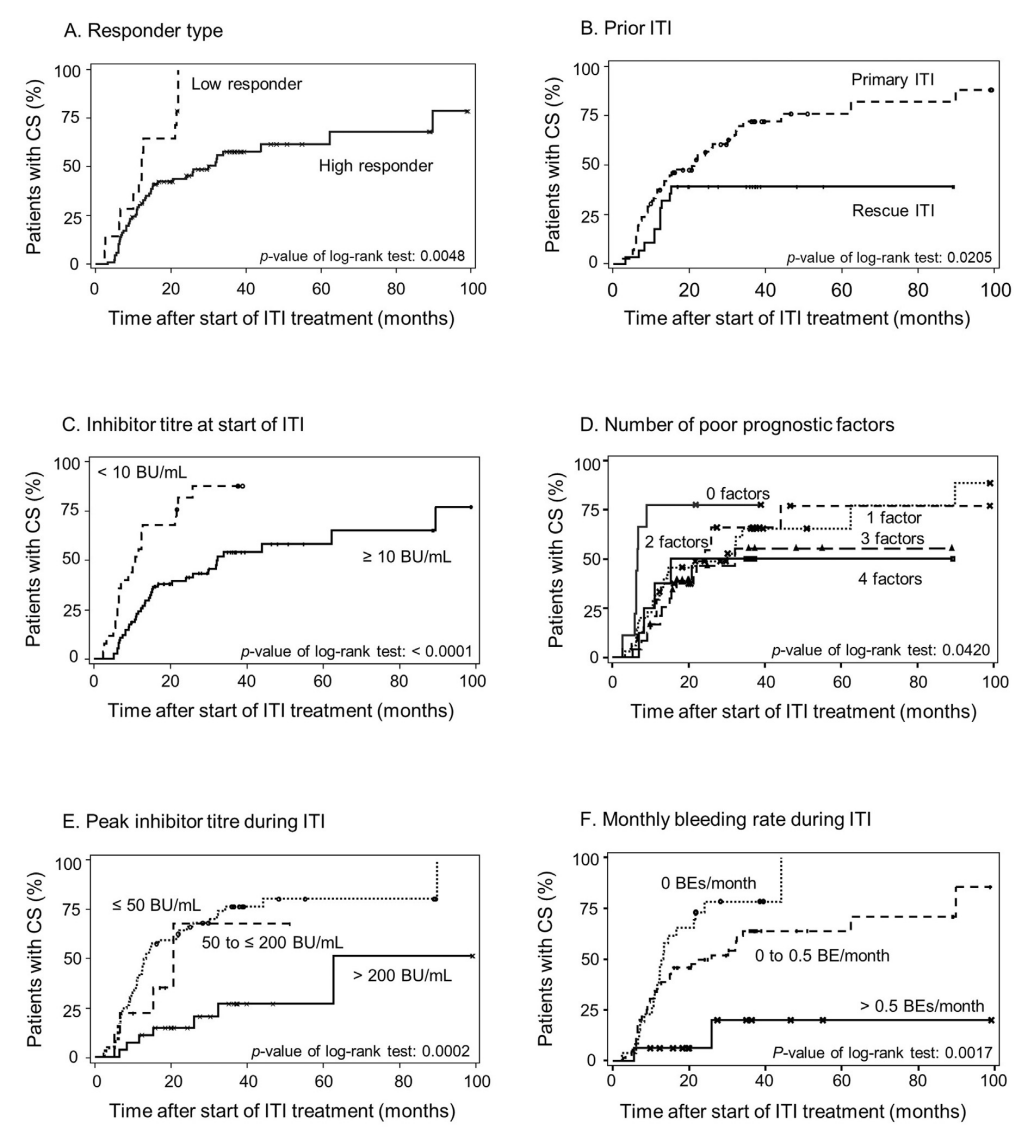
Poor prognosis factors associated with time to achievement of complete success (CS) in the overall immune tolerance induction (ITI) population ( *n* = 100). Kaplan–Meier curves showing the time-to-achievement of ITI success in relation to key variables shown to be significant in this study: ( **A** ) responder group (low vs. high *p* = 0.0048); ( **B** ) prior ITI (primary vs. rescue, *p* = 0.0205); ( **C** ) inhibitor titer at ITI start (< 10 BU/mL vs. ≥ 10 BU/mL, *p* < 0.0001); ( **D** ) number of poor prognostic factors ( *p* = 0.0420); ( **E** ) peak inhibitor titer during ITI ( *p* = 0.002); ( **F** ) monthly bleeding rate during ITI ( *p* = 0.0017).

Time to CS was significantly shorter according to responder type, prior ITI, inhibitor titer at ITI start, number of prognosis factors, peak inhibitor titer during ITI, and monthly bleeding rate during ITI ( Fig. 3 ). Time to CS was not significantly associated with time from inhibitor detection to ITI start (</≥ 2 years, *p* = 0.3956), age at ITI start (</≥ 7 years, *p* = 0.1385), dose group (high/low dose, *p* = 0.7061), or *F8* mutation type ( *p* = 0.9748).

**Fig. 4:**
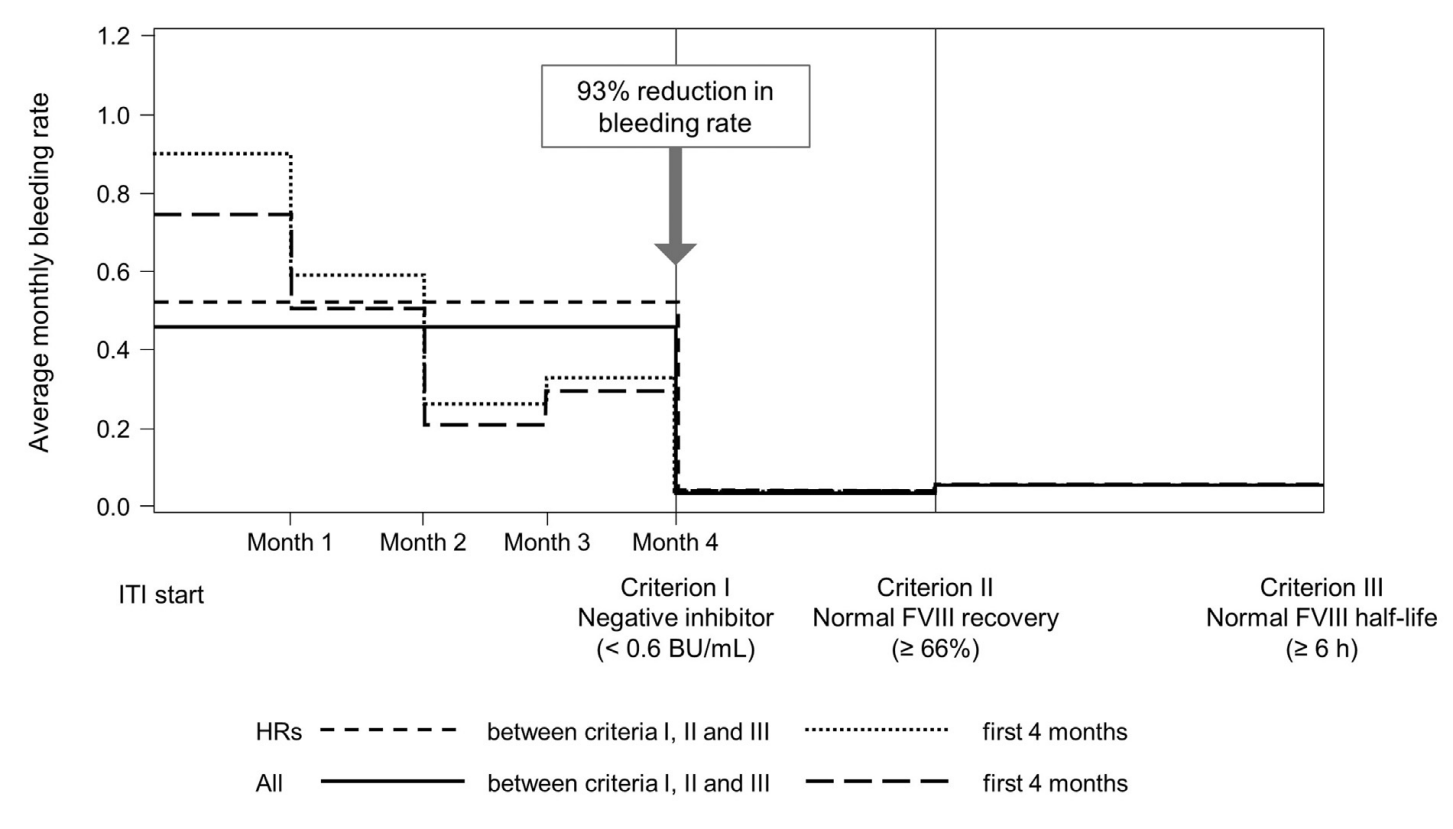
Bleeding rates before immune tolerance induction (ITI) and after achievement of criteria I, II, and III. FVIII, factor VIII.

ITI success was achieved within 9 months by 42.0% of all patients, 47.2% of primary ITI patients, and 28.6% of rescue ITI patients ( Supplementary Fig. S1A ). At 12 months, ITI success was achieved by 52.0% of all patients, 55.6% of primary ITI patients, and 42.9% of rescue ITI patients ( Fig. 4A ). In the 70 patients who achieved ITI success, 60.0% did so within 9 months and 74.3% within 1 year ( Supplementary Fig. S1B ). Three patients who achieved CS did so later than 36 months from the start of ITI, and one of these patients achieved PS later than 36 months. Two additional patients achieved PS later than 36 months.

### Bleeding Rates during ITI

In total, 769 BEs were reported in 74 of 100 patients; 24 BEs in 6 of 14 low responders and 745 BEs in 68 of 86 high responders. BEs resolved within a mean (SD) of 2.8 (3.8) days (median: 1 day) of on-demand BPA (rFVIIa or aPCC) treatment.

A statistically significant 93% reduction in mean (SD) monthly bleeding rate was observed following achievement of a negative inhibitor titer (from 0.45 [0.74] in the period before achievement to 0.03 [0.10] after achievement, *p* = 0.0002), decreasing most dramatically during the initial 4 months of ITI from 0.75 (1.38) at ITI start to 0.30 (0.85) at 4 months ( Fig. 4 ). After achieving normalized FVIII recovery and half-life, the monthly bleeding rates were 0.05 (0.13) and 0.05 (0.11), respectively, and not significantly different from the bleeding rate following achievement of a negative inhibitor titer.

In the 70 patients who achieved ITI success, the mean monthly bleeding rates were 0.39 and 0.07 before and after negative inhibitor titer, respectively, compared with 0.53 in the 30 non-success patients ( Supplementary Table S4 ).

During ITI, 14.3% of low responders and 75.6% of high responders received one or more treatment with BPAs; 7.1% of low responders and 45.3% of high responders received at least one prophylactic treatment with BPAs.

### Safety

Patients received a mean (SD) total pdFVIII/VWF dose of 128,682 (113,701) IU/kg via 1,158 (730) injections. A total of 229 ADRs/infections were reported in 43 patients and most (144 [62.9%]) were infections. Only five events in two patients were considered to be treatment related: four device-related infections in one patient and allergic dermatitis in one patient. There were 49 serious ADRs/infections in 19 patients. Only two serious ADRs/infections were considered possibly related to treatment (catheter infections of moderate intensity in the same patient).

## Discussion

This subgroup analysis reports ITI outcomes for 100 prospectively enrolled largely poor prognosis inhibitor patients in the ongoing ObsITI study undergoing ITI with octanate. This represents the largest prospective cohort with a single product in an ObsITI study to date and confirms previous findings that pdFVIII products are effective for ITI, including those from an earlier cohort of 48 patients treated with octanate in the ObsITI study. 14

Negative inhibitor titers were achieved in 71% of patients rapidly in a median of 4 months. ITI success (two or three criteria met) was achieved by 70% of the patients. PS and CS were achieved in a median of 5.55 and 11.25 months, respectively. No patients relapsed in the 12 months after achieving ITI success. The low relapse rate in this study is likely to be due at least in part to slow dose tapering and resumption of FVIII prophylaxis after achieving ITI success. However, due to the observational nature of the study, data are limited during the transition to regular prophylaxis after achievement of ITI success.

Overall ITI success rates with pdFVIII/VWF were higher in patients receiving primary ITI compared with rescue ITI, in low responders compared with high responders and in children (< 18 years) compared with adults. Factors predictive of a significantly improved ITI outcome and a significantly faster time to CS were primary ITI treatment, low responder, fewer poor prognosis factors, and a baseline inhibitor titer < 10 BU/mL. Additionally, a lower peak inhibitor titer during ITI and lower monthly bleeding rate during ITI were predictive of significantly faster time to CS. As with previous interim data 14 on 48 patients in this study, age (< 7 vs. ≥ 7 years) was not predictive of ITI success or time to achievement of ITI success. Further analysis on the influence of baseline and treatment-related factors on ITI outcomes is ongoing.

A 93% reduction in monthly bleeding rate was observed following achievement of a negative inhibitor titer and was maintained after achieving normalized IVR and half-life. In addition, 26% of patients had no bleeding events during ITI. There were only two serious ADRs in one patient that were considered related to treatment (catheter infections of moderate intensity).

The data presented here compare favorably with reports for other FVIII concentrates for ITI using similar criteria for success. 15 24 25 26 An analysis of 60 ITI patients (75% with ≥ 1 poor prognosis factor) who received a pdFVIII/VWF concentrate in the retrospective Grifols-ITI (G-ITI) study reported a CS rate (inhibitor titer < 0.6 BU/mL, FVIII recovery ≥ 66% and FVIII half-life ≥ 6 hours) of 55% in a median time of 19 months. 24 No relapses were reported during a median follow-up of 3.0 years. In a retrospective, multicenter chart review of data on 13 patients who underwent ITI with another pdFVIII/VWF, 54% of patients achieved CS in a median time of 8 months. 26 In the I-ITI study, which reported data for 66 “good risk” patients with high-titer inhibitors who were treated predominantly (90%) with rFVIII concentrates, the ITI success rate (inhibitor titer < 0.6 BU/mL, FVIII recovery ≥ 66% and FVIII half-life ≥ 6 hours or < 0.6 BU/mL with normalized recovery *or* half-life) was 74.2% in a median time of 10.6 to 15.5 months (4.6–9.2 months to achieve negative inhibitor titers); however, the 12-month relapse rate was 12.2%. 15 A retrospective study of ITI in 29 patients treated with rFVIII-Fc fusion protein reported tolerization (inhibitor titer < 0.6 BU/mL, FVIII recovery ≥ 66% and FVIII half-life ≥ 6 hours) in 44.8% patients in a median of 8.1 months, while 7 patients were still receiving ITI with rFVIII-Fc but had not achieved tolerization. 25

Given the considerable health and economic implications of FVIII inhibitors, 2 3 the time to achieve negative inhibitor titers and ITI success is of great importance to patients, physicians, healthcare providers, and wider society. Although ITI is cost-effective in the long-term, costs per patient are estimated at EUR 60,000 per month 27 and the intensive treatment regimens are demanding for patients. Rapid and sustained tolerization underpins cost-effective therapeutic inhibitor eradication, minimizing the impact of inhibitors on patients and healthcare resources alike. 28 Of the 70 patients who achieved ITI success in the present study, 60.0% did so within 9 months and 74.3% did so within 1 year of ITI start. Of 69 patients who achieved success in the G-ITI study with another pdFVIII/VWF concentrate, 23.2% did so within 9 months and 33.3% within 1 year. 28 In the study of rFVIII-Fc ITI, 53.8% of patients who achieved tolerization did so within 9 months. 25

Our analysis has some limitations due to the observational, multicenter and multinational design of the ObsITI study. There is potential for heterogeneity in patients enrolled across different centers/countries, as well as for differing “real-life” clinical practice and treatment regimens between study centers/countries. The use of local laboratories to measure FVIII inhibitor titers and FVIII pharmacokinetic parameters is a limitation as there are likely to be differences between individual laboratories. Despite the large sample size of 100 patients, the number of patients in some subgroups limits statistical analysis. For example, as the majority (73%) of patients were Caucasian, the impact of ethnicity on study outcomes could not be assessed.

Strengths of the analysis include the prospective recruitment, the use of a single product, the large sample size, the long follow-up period, and the inclusion of patients of any age with good or poor prognosis factors and treated with primary or rescue ITI. Taken together, it is likely that patients included in this analysis closely reflect clinical practice.

pdFVIII/VWF is an effective FVIII option for eradicating inhibitors in patients with hemophilia A. BEs or surgery occurring during ITI, predominately before negative inhibitor has been achieved, can be managed with BPAs, 10 although the hemostatic efficacy of BPAs is less predictive and effective than that of specific factor replacement. 22 The bispecific FIXa and FX monoclonal antibody emicizumab approved for bleeding prevention in hemophilia A patients with and without inhibitors offers a non-FVIII option. 29 Although effective in preventing BEs, emicizumab does not eradicate inhibitors and its safety profile and impact on bone and joint health are not documented in long-term studies. 1 30 Eradication of inhibitors is still a desirable goal and ITI is the only approach that currently offers this potential. 1 Eradication of inhibitors may allow the safe management of bleeds and surgery with FVIII as well as access to gene therapy. 31 The relative contributions of ITI and emicizumab in inhibitor patients will likely evolve over the coming years and this is likely to include combination therapy approaches. 1 30 32 A retrospective chart review of seven pediatric patients with severe hemophilia A who received ITI and emicizumab provided the first evidence of the feasibility and safety of a combination therapy approach. 33 Prospective studies, including the MOTIVATE study (NCT04023019, EudraCT No. 2019–003427–38), are ongoing and will compare treatment outcomes of ITI and emicizumab with ITI alone.

## Conclusion

In this ongoing study, ITI with octanate, a VWF-containing FVIII concentrate, provided a rapid, safe, and sustained eradication of FVIII inhibitors and normalization of FVIII pharmacokinetics in 70% of 100 hemophilia A patients with inhibitors and has the potential to reduce the length of ITI treatment and consequently the burden on patients and cost of ITI.
