# Supplementary material for: Immune Tolerance Induction (ITI) with a pdFVIII/VWF Concentrate (octanate) in 100 Patients in the Observational ITI (ObsITI) Study
Source: TH Open. 2022 May 26;6(2):e124–34. doi: 10.1055/s-0042-1748756 (PMC9135478; doi:10.1055/s-0042-1748756)

Supplementary Table S1 Daily FVIII dose by responder type, dosing frequency and dose

**Responder type** **Dosing frequency**	**Daily dose at ITI start, IU/kg**					
	**Low dose[a]**		**High dose[b]**		**All doses**	
	n	**Mean (SD)**	***n***	**Mean (SD)**	***N***	**Mean (SD)**
Low responders						
1x/day	1	27.8	8	109.8 (21.7)	9	100.7 (34.0)
2x/day	–	–	3	210.9 (11.1)	3	210.9 (11.1)
3x/week	–	–	1	39.0	1	39.0
3.5x/week	–	–	1	52.6	1	52.6
All frequencies	*1*	*27.8*	*13*	*123.3 (57.8)*	*14*	*116.4 (61.1)*
High responders						
1x/day	20	93.9 (24.0)	4	196.6 (32.4)	24	111.0 (46.3)
2x/day	3	123.9 (16.8)	42	266.5 (77.3)	45	257.0 (82.9)
2x/week	1	16.8	–	–	1	16.8
3x/week	9	28.1 (7.5)	–	–	9	28.1 (7.5)
4x/week	1	44.0	–	–	1	44.0
3.5x/week	6	65.3 (27.9)	–	–	6	65.3 (27.9)
All frequencies	*40*	*73.9 (37.5)*	*46*	*260.5 (76.9)*	*86*	*173.7 (112.0)*
All patients						
1x/day	21	90.7 (27.5)	12	138.7 (49.1)	33	108.2 (43.0)
2x/day	3	123.9 (16.8)	45	262.8 (75.9)	48	254.2 (81.0)
2x/week	1	16.8	–	–	1	16.8
3x/week	9	28.1 (7.5)	1	39.0	10	29.2 (7.9)
4x/week	1	44.0	–	–	1	44.0
3.5x/week	6	65.3 (27.9)	1	52.6	7	63.5 (25.9)
All frequencies	*41*	*72.7 (37.7)*	*59*	*230.2 (92.5)*	*100*	*165.7 (108.0)*

[a]Low dose: < 150 IU FVIII/kg/day for high responders, < 50 IU FVIII/kg either daily or every other day for low responders.

[b]High dose: ≥ 150 IU FVIII/kg/day for high responders, ≥ 50 IU FVIII/kg either daily or every other day for low responders.

Abbreviations: ITI, immune tolerance induction; IU, international units; SD, standard deviation.

Supplementary Table S2 Daily FVIII dose by responder type, dosing frequency and dose in patients who achieved ITI success

Responder type<br>Dosing frequency	Daily dose at ITI start in patients who achieved ITI success[a], IU/kg					
	Low dose[b]		High dose[c]		All doses	
	n	**Mean (SD)**	***n***	**Mean (SD)**	***N***	**Mean (SD)**
Low responders						
1x/day	1	27.8	7	111.7 (22.6)	8	101.2 (36.3)
2x/day	–	–	3	210.9 (11.1)	3	210.9 (11.1)
3x/week	–	–	1	39.0	1	39.0
3.5x/week	–	–	1	52.6	1	52.6
All frequencies	*1*	*27.8*	*12*	*125.5 (59.8)*	*13*	*118.0 (63.4)*
High responders						
1x/day	14	90.5 (25.2)	3	184.6 (26.7)	17	107.1 (44.4)
2x/day	1	117.7	25	268.7 (86.3)	26	262.9 (89.6)
2x/week	–	–	–	–	–	–
3x/week	8	29.2 (7.2)	–	–	8	29.2 (7.2)
4x/week	–	–	–	–	–	–
3.5x/week	6	65.3 (27.9)	–	–	6	65.3 (27.9)
All frequencies	*29*	*69.3 (34.9)*	*28*	*259.7 (85.9)*	*57*	162.8 (115.7)
All patients						
1x/day	15	86.3 (29.2)	10	133.6 (41.7)	25	105.2 (41.3)
2x/day	1	117.7	28	262.5 (83.4)	29	257.5 (86.2)
2x/week	–	–	–	–	–	–
3x/week	8	29.2 (7.2)	1	39.0	9	30.3 (7.5)
4x/week	–	–	–	–	–	–
3.5x/week	6	65.3 (27.9)	1	52.6	7	63.5 (25.9)
All frequencies	*30*	*67.9 (35.1)*	*40*	*219.5 (100.0)*	*70*	*154.5 (109.0)*

[a]Success = Complete success or partial success.

[b]Low dose: < 150 IU FVIII/kg/day for high responders, < 50 IU FVIII/kg either daily or every other day for low responders.

[c]High dose: ≥ 150 IU FVIII/kg/day for high responders, ≥ 50 IU FVIII/kg either daily or every other day for low responders.

Abbreviations: ITI, immune tolerance induction; IU, international units; SD, standard deviation.

Supplementary Table S3 Daily FVIII dose by responder type, dosing frequency and dose in patients who did not achieve ITI success

**Responder type**<br>**Dosing frequency**	**Daily dose at ITI start in patients who did not achieve ITI success[a], IU/kg**					
	**Low dose[b]**		**High dose[c]**		**All doses**	
	n	**Mean (SD)**	***n***	**Mean (SD)**	***N***	**Mean (SD)**
Low responders						
1x/day	–		1	96.2	1	96.2
2x/day	–	–	–	–	–	–
3x/week	–	–	–	–	–	–
3.5x/week	–	–	–	–	–	–
All frequencies	–	–	1	96.2	*1*	*96.2*
High responders						
1x/day	6	101.7 (20.9)	1	232.6	7	120.4 (53.0)
2x/day	2	127.0 (22.5)	17	263.3 (64.1)	19	249.0 (74.3)
2x/week	1	16.8	–	–	1	16.8
3x/week	1	19.3	–	–	1	19.3
4x/week	1	44.0	–	–	1	44.0
3.5x/week	–	–	–	–	–	–
All frequencies	*11*	*85.8 (43.0)*	*18*	*261.6 (62.6)*	*29*	*195.0 (102.8)*
All patients						
1x/day	6	101.7 (20.9)	2	164.4 (96.5)	8	117.4 (49.8)
2x/day	2	127.0 (22.5)	17	263.3 (64.1)	19	249.0 (74.3)
2x/week	1	16.8	–	–	1	16.8
3x/week	1	19.3	–	–	1	19.3
4x/week	1	44.0	–	–	1	44.0
3.5x/week	–	–	–	–	–	–
All frequencies	*11*	*85.8 (43.0)*	*19*	*252.9 (71.7)*	*30*	*191.7 (102.6)*

[a]Success = Complete success or partial success.

[b]Low dose: < 150 IU FVIII/kg/day for high responders, < 50 IU FVIII/kg either daily or every other day for low responders.

[c]High dose: ≥ 150 IU FVIII/kg/day for high responders, ≥ 50 IU FVIII/kg either daily or every other day for low responders.

Abbreviations: ITI, immune tolerance induction; IU, international units; SD, standard deviation.

Supplementary Table S4 Mean (SD) monthly bleeding rates during ITI in patients who achieved success and non-success patients

	**Non-success patients (*n* = 30)**	**ITI success[a] patients (*n* = 70)**	
**BE severity** **Dose group**		**Prior to negative inhibitor**	**After negative inhibitor**
All BEs			
All patients	0.53 (0.88)	0.39 (0.65)	0.07 (0.13)
Low-dose patients[b]	0.38 (0.35)	0.34 (0.42)	0.09 (0.18)
High-dose patients[c]	0.61 (1.07)	0.43 (0.79)	0.05 (0.08)
Mild BEs			
All patients	0.15 (0.18)	0.35 (0.60)	0.07 (0.11)
Low-dose patients[b]	0.07 (0.04)	0.18 (0.24)	0.07 (0.13)
High-dose patients[c]	0.21 (0.22)	0.52 (0.78)	0.06 (0.07)
Moderate BEs			
All patients	0.34 (0.52)	0.30 (0.37)	0.04 (0.05)
Low-dose patients[b]	0.23 (0.21)	0.33 (0.34)	0.03 (0.03)
High-dose patients[c]	0.41 (0.65)	0.27 (0.39)	0.04 (0.06)
Severe BEs			
All patients	0.19 (0.30)	0.12 (0.14)	0.01 (0.02)
Low-dose patients[b]	0.16 (0.15)	0.12 (0.10)	0.01 (0.01)
High-dose patients[c]	0.20 (0.36)	0.12 (0.17)	0.02 (0.02)

[a]Complete success or partial success.

[b]Low dose: < 150 IU/kg/day for high responders, < 50 IU/kg either daily or every other day for low responders.

[c]High dose: ≥ 150 IU/kg/day for high responders, ≥ 50 IU/kg either daily or every other day for low responders.

Abbreviations: BE, bleeding episode; ITI, immune tolerance induction.

Supplementary Figure S1 Proportion of patients achieving ITI success by 9 or 12 months or at any time from start of ITI. (A) overall population (n = 100), (B) patients who achieved success (n = 70).

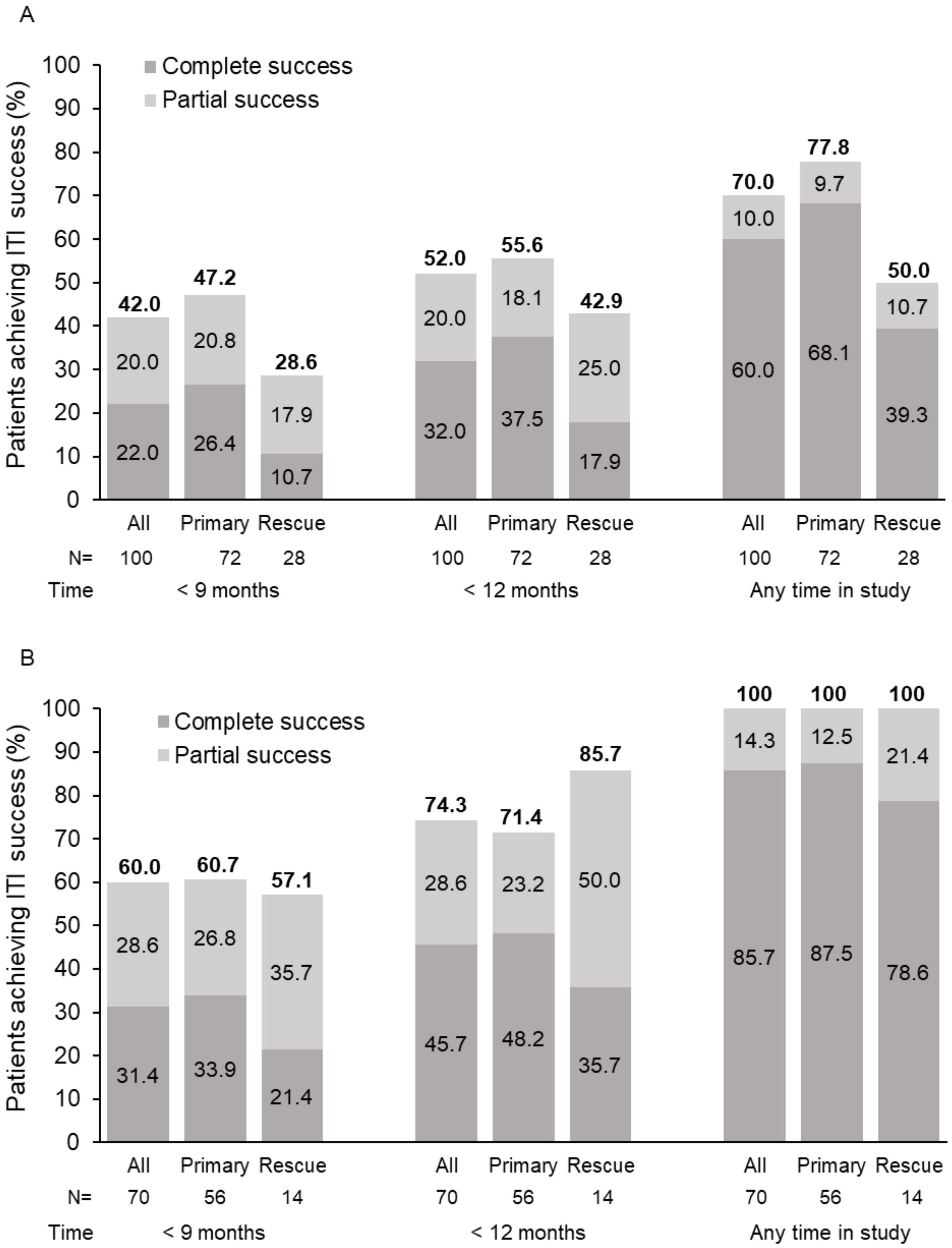

Supplement: Supplementary file 1 — Supplementary Material [file 10-1055-s-0042-1748756-s210082.pdf]
